# β-PIX controls intracellular viscoelasticity to regulate lung cancer cell migration

**DOI:** 10.1111/jcmm.12441

**Published:** 2015-02-16

**Authors:** Helen Wenshin Yu, Yin-Quan Chen, Chi-Ming Huang, Ching-Yi Liu, Arthur Chiou, Yang-Kao Wang, Ming-Jer Tang, Jean-Cheng Kuo

**Affiliations:** a Institute of Biochemistry and Molecular Biology, National Yang-Ming UniversityTaipei, Taiwan; b Institute of Biophotonics, National Yang-Ming UniversityTaipei, Taiwan; c Institute of Basic Medical Sciences, National Cheng Kung UniversityTainan, Taiwan; d Department of Physiology, National Cheng Kung UniversityTainan, Taiwan; e Biophotonics & Molecular Imaging Research Center, National Yang-Ming UniversityTaipei, Taiwan; f Department of Cell Biology and Anatomy, National Cheng Kung UniversityTainan, Taiwan; g Center for Neurotrauma and Neuroregeneration, Taipei Medical UniversityTaipei, Taiwan

**Keywords:** non-small cell lung adenocarcinoma cells, stress fibres, focal adhesions, β-PIX, viscoelasticity, cell stiffness, cell migration

## Abstract

Cancer metastasis occurs *via* a progress involving abnormal cell migration. Cell migration, a dynamic physical process, is controlled by the cytoskeletal system, which includes the dynamics of actin organization and cellular adhesive organelles, focal adhesions (FAs). However, it is not known whether the organization of actin cytoskeletal system has a regulatory role in the physiologically relevant aspects of cancer metastasis. In the present studies, it was found that lung adenocarcinoma cells isolated from the secondary lung cancer of the lymph nodes, H1299 cells, show specific dynamics in terms of the actin cytoskeleton and FAs. This results in a higher level of mobility and this is regulated by an immature FA component, β-PIX (PAK-interacting exchange factor-β). In H1299 cells, β-PIX's activity was found not to be down-regulated by sequestration onto stress fibres, as the cells did not bundle actin filaments into stress fibres. Thus, β-PIX mainly remained localized at FAs, which allowed maturation of nascent adhesions into focal complexes; this resulted in actin polymerization, increased actin network integrity, changes in the intracellular microrheology at the peripheral of the cell, and cell polarity, which in turn regulated cell migration. Perturbation of β-PIX caused an inhibition of cell migration, including migration velocity, accumulated distance and directional persistence. Our results demonstrate the importance of β-PIX to the regulation of high mobility of lung adenocarcinoma cell line H1299 and that this occurs *via* regulation of FA dynamics, changes in actin cytoskeleton organization and cell polarity.

## Introduction

Cancer cells undergo migration and invasion to different positions within tissues. Cancer cells exhibit a mesenchymal-type movement or amoeboid-like mode to change cell shape and migrate 1. Cell migration is mediated *via* various cell-scale dynamic macromolecular ensembles. The physical phenomena that drive cell migration include pushing and pulling forces, as well as changes in elasticity and viscosity 2–6. These events are spatially and temporally controlled by the cytoskeletal system, which includes the dynamics of actin filaments and changes in the cellular adhesive organelles, focal adhesions (FAs). For most cells in most environments, constitutive mesenchymal-type migration is accomplished *via* a repetitive sequence of the following four-step cycles 7–9. At the beginning, along the leading edge in the direction of migration, the extension of a lamellipodium protrusion is characterized by actin polymerization into dense actin networks. Subsequently, the protrusion adheres to the extracellular matrix (ECM) *via* nascent FAs, which are then matured into focal complexes by Rac1-mediated signals. In the lamella, the convergence zone, and the central cell area, myosin II-mediated contractile forces are transduced along the bundles of actin filaments (stress fibres) to the ECM *via* mature FAs, thereby pulling the cell body forward. Finally, myosin II-driven contractile forces pull and detach FAs at the trailing end of the cell from the ECM. Importantly, the dynamics of actin networks and FAs must be orchestrated in a precise spatial and temporal order to direct cell movement 10.

Focal adhesions, an adhesive organelle, start to form when the central component, the integrin receptor, is activated by engagement with the ECM, which is then followed by the subsequent recruitment of a series of FA-associated proteins, including various structural, signalling and scaffold proteins, to connect with the actin cytoskeleton 11–15. A process whereby FAs grow in size, called FA maturation, simultaneously changes the composition of FAs such that specific components are able to transduce distinct signals downstream of integrin engagement 16–18. The components present in immature FAs mediate FA formation and the extension of lamellipodia protrusion, which suggests that the proteins present in FAs may control remodelling of actin cytoskeleton networks and as a result control the migration of cancer cells.

Rho guanine nucleotide exchange factor (GEF) 7, β-PIX (PAK-interacting exchange factor-β; β-PIX), has been identified as a GEF protein with activity towards Rac1 and Cdc42 19,20, proteins that are known to promote the extension of lamellipodia protrusion by polymerizing actin filaments 17 and cell polarization on directed cell migration 21 respectively. The activity of β-PIX is negatively regulated on association with myosin II heavy chains within stress fibres 22 and is switched on when myosin II ATPase is inhibited, which disrupts the structures of stress fibres. This releases β-PIX which then localizes to FAs 17,22. The accumulation of β-PIX within FAs promotes an increase in Rac1 activity, higher levels of lamellipodia formation and increased cell motility 17,23. Some studies have reported that overexpression of β-PIX occurs in many cancers 24,25 and such overexpression would seem to have an important effect on lung cancer metastasis 26.

In the present study, we examined whether β-PIX controls the mobility of non-small cell lung adenocarcinoma cells and if so, how. We used the non-small cell lung adenocarcinoma cell line H1299 as the model system, as we found that, in comparison with the non-small cell lung adenocarcinoma cells isolated from the primary lung cancer in the lung, H1299 cells, which were isolated from the secondary lung cancer of the lymph nodes, contain a specific organization of actin cytoskeleton that does not form bundled actin filaments (stress fibres); furthermore, these cells are highly mobile. By examining the effects of β-PIX on the organization of FAs and the actin cytoskeleton, we have confirmed that β-PIX mediates the regulation of focal complexes formation, controls of the formation of actin filaments networks, controls intracellular viscoelasticity, enhances cell polarity and directs lung cancer cell migration.

## Materials and methods

### Cells

H1299 and A549 cells (human non-small cell lung adenocarcinoma cell lines) were gifts from Prof. R.-H. Chen's laboratory (Academia Sinica, Taipei, Taiwan), and were maintained in DMEM-high glucose (Invitrogen, Carlsbad, California, USA) supplemented with 10% FBS (Invitrogen) and 1% antibiotic solution (penicillin and streptomycin; Invitrogen) under 5% CO_2_. CL1-0 and CL1-5 cells (human non-small cell lung adenocarcinoma cell lines were provided by Prof. P.-C. Yang, National Taiwan University, Taipei, Taiwan) were maintained in RPMI-1640 medium (Gibco) supplemented with 10% FBS (Invitrogen) and 1% antibiotic solution (penicillin and streptomycin; Invitrogen) under 5% CO_2_. H1299 cells stably expressing β-PIX shRNA were generated using a lentiviral shRNA system according to manufacturer's instructions (the National RNAi Core Facility Platform) and selected with puromycin. Transfection was carried out using Turbofect reagent (ThermoFisher, Waltham, Massachusetts, USA). For all experiments, cells were seeded on 10 μg/ml fibronectin (homo)-coated coverslips or plates. The control cells used for all the blebbistatin studies were treated with the same volume of DMSO.

### Plasmids, antibodies and reagents

#### shRNAs

Expression silencing of β-PIX was achieved using the pLKO.1 construct obtained from the National RNAi Core Facility Platform (TRCN0000047596). The target sequence is GAAGTTAAGTTCAGCAAACAT.

#### Expression constructs

To derive the GFP-C1-β-PIX (mouse), full-length β-PIX cDNA was PCR amplified from the template Flag-ECFP-β-PIX (Addgene, Cambridge, Massachusetts, USA) and cloned into the pGFP-C1 vector (Clontech, Mountain View, California, USA).

#### Antibodies

The sources of antibodies and the dilutions used in experiments were as follows: rabbit anti-β-PIX (Millipore 07-1450, Darmstadt, Germany; dilution for Western blotting: 1/1000; dilution for immunofluorescence: 1/500); mouse anti-paxillin (BD Bioscience 610052, San Jose, California, USA; dilution for immunofluorescence: 1/1000); mouse anti-GAPDH (GeneTex GTX627408, Hsinchu, Taiwan; dilution for Western blotting: 1/1000); DNase I (Sigma-Aldrich SAB2702031, Saint Louis, Missouri, USA; dilution for immunofluorescence: 1:1000); Alexa Fuor 488-antimouse IgG (Jackson ImmunoResearch 715-546-151/J1, West Grove, Philadelphia, USA; dilution for immunofluorescence: 1/300); Alexa Fuor 568-antimouse IgG (Invitrogen A11031; dilution for immunofluorescence: 1/300); Alexa Fuor 568-anti-rabbit IgG (Invitrogen A11036; dilution for immunofluorescence: 1/300); Alexa Fuor 647-antimouse IgG (Jackson ImmunoResearch 715-606-151/J1; dilution for immunofluorescence: 1/300). Alexa Fluor 488 phalloidin (A12379; dilution for immunofluorescence: 1/400) and Alexa Fluor 568 phalloidin (A12380; dilution for immunofluorescence: 1/400) were obtained from Invitrogen. HRP-AffiniPure goat antimouse IgG (115-035-174; dilution for Western blotting: 1/5000) and HRP-AffiniPure goat anti-rabbit IgG (211-032-171; dilution for Western blotting: 1/5000) were obtained from Jackson ImmunoResearch.

#### Reagents

Blebbistatin was obtained from Toronto Research Chemical (TRC).

### Immunofluorescence analysis

Cells were fixed and immunostained using the method described previously 17. For phalloidin and/or DNase I staining, cells were mounted on slides with Dako mounting medium. The epi-flourescence images were obtained using a 100X 1.30NA objective lens on a LEICADMRBE microscope (Wetzlar, Germany) equipped with a Coolsnap EZ CCD camera (Photometrics, Tucson, Arizona, USA). To calculate the ratio of F-actin to G-actin, the images were captured using the same exposure time, and the integrated intensity of phalloidin and DNase I was quantified using Metamorph image analysis software (Molecular Device, Sunnyvale, California, USA). To capture the spinning disc confocal images, the slides with cells were imaged with a 100X 1.49NA (Immersion) Plan objective lens (Nikon, Tokyo, Japan) using the *iLas* multi-modal of TIRF (Roper Scientific, Evry Cedex, France)/spinning disc confocal (CSUX1, Yokogawa, Tokyo, Japan) microscope system equipped with an EMCCD camera (ProEM, Princeton, Instruments, Trenton, New Jersey, USA). For phalloidin, β-PIX, and paxillin staining, cells were mounted on slides with PBS containing N-propyl gallate; then epi-fluorescence (phalloidin) and TIRF images (β-PIX and paxillin) were obtained with a 100X 1.49NA (Immersion) Plan objective lens (Nikon) using the *iLas* multi-modal of TIRF (Roper)/spinning disc confocal (CSUX1, Yokogawa) microscope system equipped with a Coolsnap HQ2 CCD camera (Photometrics).

### Time-lapse microscopy

To analyse the dynamics of F-actin and paxillin, H1299 cells co-expressing F-tractin-GFP and mApple-paxillin were imaged by epi-fluorescence and TIRF, respectively, using a 100X 1.49NA (Immersion) Plan objective lens (Nikon) on the *iLas* multi-modal of TIRF (Roper)/spinning disc confocal (CSUX1, Yokogawa) microscope system. The epi-fluorescence and TIRF images were captured using a Coolsnap HQ2 CCD camera (Photometrics).

### Random migration analysis

Cells were plated on six-well plates in the regular culture medium for 16 hrs, and then placed in a temperature-controlled and CO_2_-controlled chamber of a microscope (Axio Observer.Z1; Zeiss, Jena, Germany). Time-lapse images were obtained at 5 min intervals over 180 min using a 10X 0.25NA objective lens (Zeiss) using this microscope system with an AxioCamMR3 CCD camera. To calculate the motility parameters, including velocity, accumulated distance and directional persistence, the trajectories of the cells were recorded by tracing the geometric centre of nucleus for each frame using the Metamorph image analysis software (Molecular Device), and were then presented graphically using the Excel software (Microsoft, Albuquerque, New Mexico, USA). The average velocity was calculated as the ratio of the net distance (*i.e*. the length of the displacement vector from the initial to the end-points) over a 180 min period of migration. The accumulated distance travelled was determined as the total length of the trajectory over a 180 min period. Directional persistence was calculated as a ratio of the net distance to the total length of the trajectory (accumulated distance).

### Video particle tracking microrheology

Non-silencing and β-PIX-silencing H1299 cells (7 × 10^6^ cells) were seeded on 10 cm tissue culture dish, and were then injected with 20 μl carboxylated polystyrene particles (Invitrogen, fluorescence excitation/admission peaks: 580 nm/605 nm, diameter = 100 nm, concentration: 2.7 × 10^8^ particles/μl) using a biolistic particle delivery system (PDS-100; Bio-Rad, Hercules, California, USA; pressure 450 psi). Cells were then washed with PBS twice and incubated in culture medium. After 4 hrs of incubation, cells were trypsinized and plated on fibronectin-coated 22 mm^2^ coverslips (1 × 10^5^ cells/coverslip). The coverslips were placed in a temperature-controlled and CO_2_-controlled chamber (Tokai Hit, Shizuoka-ken, Japan) of an inverted microscope (Nikon Eclipse Ti). The two-dimensional Brownian motion of intracellular fluorescence beads was tracked and recorded with an inverted epi-fluorescence microscope (Nikon Eclipse Ti) equipped with an oil-immersion objective (Nikon, 100X/N.A. 1.45) and a CMOS camera (OHCA-Flash 4.0; Hamamatsu, Hamamatsu City, Japan); the sampling rate was 100 Hz for 10 sec. with an image resolution of 260 nm/pixel, and 512 × 512 pixels per frame.

The two-dimensional Brownian motion of the intracellular fluorescence beads was analysed using customized MatLab Program by Kathryn Osterday and Prof. Juan Carlos del Alamo (Department of Mechanical and Aerospace Engineering; UCSD) and Dr. Yin-Quan Chen (Institute of Biophotonics; National Yang-Ming University, Taiwan). Based on the trajectory [*x*(*t*) and *y*(*t*), as a function of time (*t*)] of each particle, the mean squared displacement (MSD) (<Δr2(τ)> = <[*x*(*t*+τ) − *x*(*t*)]2 + [*y*(*t*+τ) − *y*(*t*)]2>, where τ is the time lag and *t* is the elapsed time) of each particle was calculated. The intracellular viscoelastic properties (the elastic modulus G′ and the viscous modulus G″) were obtained from the MSD based on a pseudo-Stokes Einstein equation 27,28. We limited our analysis to the range of time lags 0.01 s < τ < 1 s. To exclude the particles that exhibited non-Brownian motion, only the particle trajectories consistent with 0 < α < 1, where ‘α’ is the exponent in the expression MSD = <Δr^2^(τ)> = Aτ^α^, were retained for the calculation of G′ and G″.

### Measurement of cell stiffness by AFM indentation

Cells were plated at the density of 3 × 10^3^ cells/cm^2^, and cellular stiffness on top of the nucleus and the cytosol was measured using atomic force microscopy (AFM) indentation method 29–31 by the JPK NanoWizard II AFM system (JPK Instruments, Berlin, Germany). The cantilever was the tipless cantilever (ARROW-TL1-50; NanoWorld, Neuchatel, Switezerland), which conjugated with a polystyrene bead (with diameter of 5 μm). Before each measurement, cantilever was calibrated and possessed a mean spring constants ranging from 0.01 to 0.03 nN/nm. For stiffness measurements, 1 nN indentation was applied to each cell. The indentation depth was about 0.5 μm to avoid the substratum effect. The approaching speed of the cantilever to cell surface was set at 1 μm/sec. Force-distance curves were collected by landing the bead on region of interests and processed using JPK IP software. To extract the mechanical properties of cells from force-distance curves, the data were analysed by the Hertz model.

### Statistical analysis

Statistical significance was measured by a two-tailed Student's *t*-test.

## Results

### Actin filaments in H1299 lung adenocarcinoma cells isolated from a secondary lung cancer of the lymph nodes are not bundled into stress fibres

**Figure 1:**
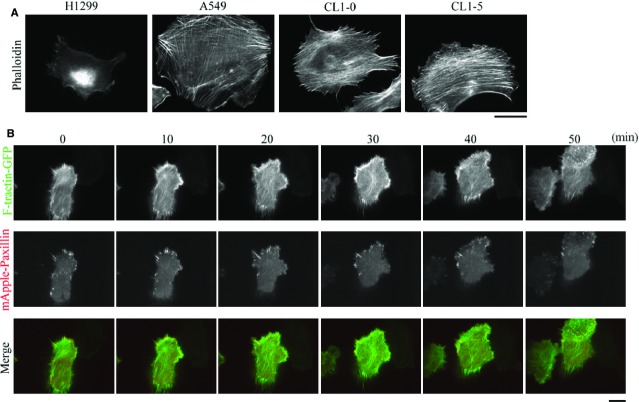
Staining for F-actin in non-small cell lung adenocarcinoma cells reveals that the cells isolated from a secondary cancer of the lymph nodes, namely H1299 cells, do not form bundled actin filaments (stress fibres) during cell migration. (A) CL1-0, CL1-5, A549 and H1299 cells were immunostained with fluorescent phalloidin, to localize F-actin; bar 15 μm. (B) Time-lapse TIRF images of H1299 cells expressing F-tractin-GFP and mApple-paxillin showing the dynamics of F-actin and FAs, respectively; bar 15 μm.

To understand how the mobility of non-small cell lung adenocarcinoma cells is correlated with the dynamics of F-actin, we first examined the F-actin distribution in non-small cell lung adenocarcinoma cells that had been isolated from primary lung cancer of the lung, including A549, CL1-0 and CL1-5 cells, and in the non-small cell lung adenocarcinoma cells that had been isolated from the secondary lung cancer of the lymph nodes, H1299 cells. Immunolocalization of F-actin showed that there was formation of bundled actin filaments (stress fibres) in A549, CL1-0 and CL1-5 cells, but this was not the case for H1299 cells (Fig.1A). To further examine the dynamics of actin filaments and FAs in migrating H1299 cells, cells were cotransfected with F-tractin-GFP (inositol 1,4,5-Trisphosphate 3-Kinase A N66 actin binding domain fused to GFP 32) and mApple-paxillin to simultaneously observe the patterns of F-actin and FAs using epi-fluorescence and TIRF microscopy, respectively. On the basis of the time-lapse images (Video S1), we observed the presence in cells of an F-actin-rich lamellipodium, together with the formation of nascent adhesions that extended to the cell edge. This was followed by FA maturation and disassembly, which resulted in cell body movement and retraction. It was clear that, during the migration cycle, H1299 cells did not produce bundled actin filaments (stress fibres; Fig.1B), which suggests that suppression of bundled actin filaments (stress fibres) formation is able to promote the mobility of non-small cell lung adenocarcinoma cells.

### β-PIX regulates directed cell migration in H1299 cells

**Figure 2:**
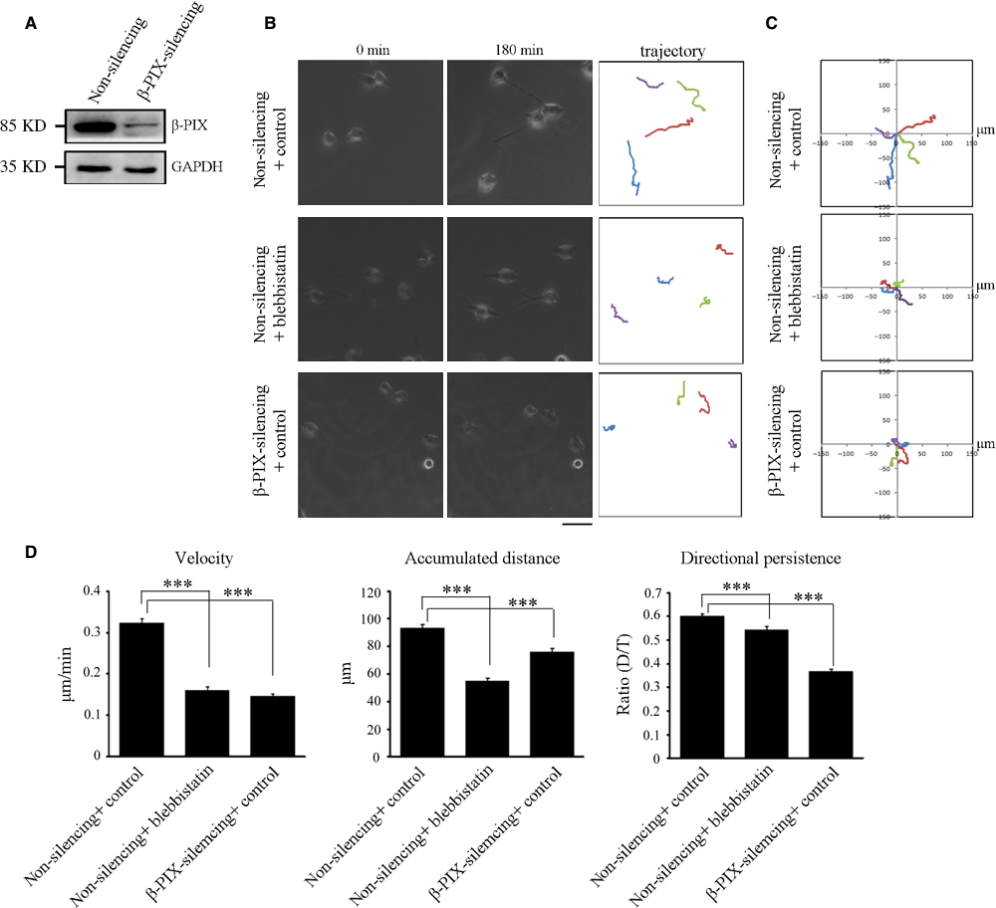
Myosin II ATPase inhibition results in similar effects to that obtained when β-PIX is silenced in terms of the regulation of directed cell migration. (A) Cell lysate from non-silencing and β-PIX-silencing H1299 cells were analysed by Western blotting. (B) The migratory behaviour of non-silencing and β-PIX-silencing H1299 cells treated with blebbistatin (50 μM) or control (DMSO) for 2 hrs. Cells were plated for 16 hrs, and then monitored for 180 min (left and middle) Images at 0 and 180 min, respectively. (right) These images delineated the trajectory of each cell during the 180 min period; bar 50 μm. Coloured lines were used to distinguish overlapped cell trajectories. (C) Analysis of migration trajectories. The trajectories of representative cells are plotted. The origins of migration are superimposed at (0, 0). (D) The migration parameters were calculated as described in Materials and methods. Data are mean ± SEM (non-silencing H1299 cells treated with control, *n* = 353 cells; non-silencing H1299 cells treated with blebbistatin, *n* = 307 cells; β-PIX-silencing H1299 cells treated with control, *n* = 144 cells). ***P < 0.001.

The mobility of cells is known to be up-regulated by the Rac1 GEF protein, β-PIX 17. β-PIX has been demonstrated to be inactivated by sequestration by bundled actin filaments (stress fibres) 22, and to be switched on when myosin II ATPase is inhibited. Such inhibition disrupts stress fibres and releases β-PIX from stress fibres allowing take-up by FAs 17,22. On the basis of the above, we examined whether β-PIX functions as a regulator of H1299 cell motility. Non-silencing and β-PIX-silencing H1299 cells were generated (Fig.2A) and subjected to random migration analysis. We first analysed the trajectory of each individual cell over a 180 min migration period by tracing its geometric centre of each cell's nucleus using time-lapse images (Video S2), and found that, in comparison with non-silencing cells, β-PIX-silencing cells displayed a much shorter net translocation, and changed their migration direction much more frequently (Fig.2B, right). These findings are more clearly visualized when presented as trajectories on window plots (Fig.2C). We also calculated the parameters shown in Figure2D, which revealed that higher levels of β-PIX expression promoted the migration velocity, the accumulated distances over a 180 min time period and the directional persistence of cells. Therefore, it would seem that β-PIX plays a crucial role in enhancing cell motility and cell polarity, thereby regulating the directed migration of H1299 cells.

As myosin II ATPase inhibition promotes lamellipodia extension and contributes to fibroblast cell migration 17, we next examine whether myosin II ATPase inhibition regulates cell motility in H1299 cells, which do not form bundled actin filaments (stress fibres). In random migration analysis (Video S2), we found that treatment with blebbistatin, a myosin II ATPase inhibitor, in non-silencing H1299 cells resulted in a much shorter net translocation and accumulated distance compared to non-silencing cells treated with DMSO (Fig.2B, right). In addition, non-silencing cells treated with blebbistatin showed similar effects as β-PIX-silencing, namely more frequent changes in migration direction in comparison with control cells (Fig.2B and C). The motility parameters indicated that myosin II ATPase inhibition suppressed the velocity of cell migration, reduced the accumulated distance, and produced a reduction in the directional persistence of cells (Fig.2D). These findings reveal that, in H1299 cells, β-PIX and myosin II ATPase activity promote cell migration velocity, accumulated distance and directional persistence. Taken together, our findings suggest that inhibition of myosin II ATPase activity in the non-small cell lung adenocarcinoma cells isolated from the lymph nodes shows different effects as those that occur in fibroblasts, where myosin II ATPase inhibition promotes mobile capability through activating β-PIX 17, and these effects in H1299 cells might be because of the facts that they lack bundled actin filaments (stress fibres).

### β-PIX localizes at paxillin-marked adhesions and drives focal complexes formation

**Figure 3:**
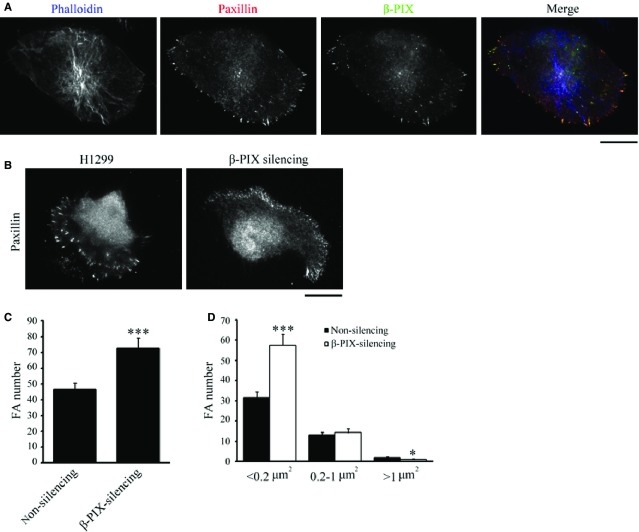
β-PIX is mainly localized at FAs and regulates FA dynamics. (A) H1299 cells were immunostained to localize F-actin (blue; phalloidin), paxillin (red; as a focal adhesion marker), and β-PIX (green); bar 15 μm. (B–D) Non-silencing and β-PIX-silencing H1299 cells were immunostained with paxillin, to localize FAs, and imaged by TIRF microscopy (B); bar 15 μm. The number (C) and size distribution (D) of segmented paxillin-marked adhesions within the cells. Data are mean ± SEM (non-silencing, *n* = 1118 FAs/24 cells; β-PIX-silencing, *n* = 1744 FAs/24 cells). ***P < 0.001, *P < 0.05, in comparison with non-silencing cells.

As actin filaments are not bundled into stress fibres in H1299 cells, we examined whether β-PIX is localized at FAs rather than with F-actin. Immunolocalization of β-PIX, paxillin and phalloidin indicated that β-PIX is localized at paxillin-marked adhesions, not with F-actin (Fig.3A). Next, we determined the role of β-PIX in adhesions maturation of H1299 cells. Immunolocalization of paxillin in H1299 cells when β-PIX was silenced. We found that there was a significant increase in the number of paxillin-marked adhesions compared to non-silencing cells (Fig.3B and C). Surprisingly, quantitative analysis of the paxillin-marked adhesions identified a unique feature of H1299 cells, namely that only around 1.8% of adhesions were bigger than 1 μm^2^. Thus, there is an absence of mature, big, FAs in H1299 cells, probably because of the absence of stress fibres to transduce bundled contractile force in these cells. Furthermore, another effect of silencing β-PIX was to significantly increase the number of small adhesions (<0.2 μm^2^; Fig.3D), which suggests that, in H1299 cells, β-PIX mediates the process of maturating nascent adhesion into focal complexes.

### β-PIX regulates actin cytoskeleton architecture at the cortex of H1299 cells

**Figure 4:**
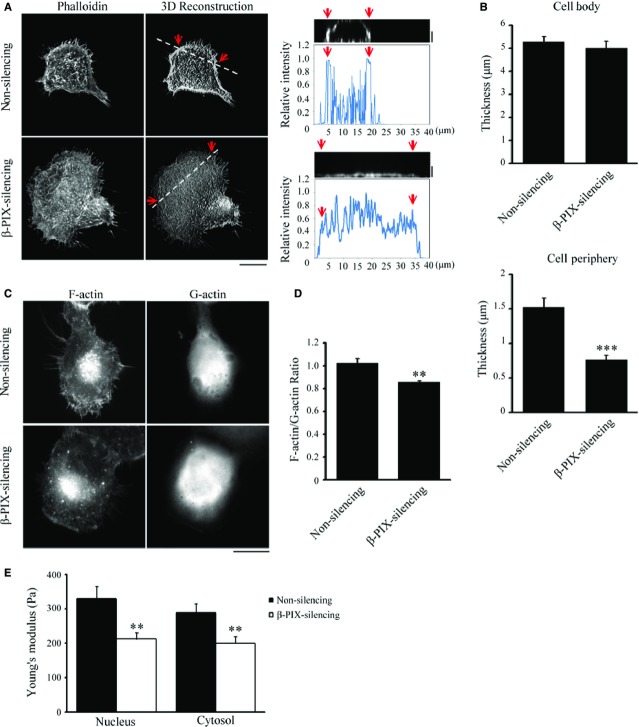
β-PIX promotes actin polymerization and regulates actin cytoskeletal architecture. (A, left) Confocal images of non-silencing and β-PIX-silencing H1299 cells stained with fluorescent phalloidin to visualize F-actin. (Middle) 3D reconstruction taken from the z-series of confocal images of non-silencing and β-PIX-silencing H1299 cells stained with fluorescent phalloidin at every 0.2 μm depth from top to bottom; bar 15 μm. (Right) The side view image and relative intensity taken along the line highlighted in the 3D reconstruction image with the edge marked with red arrows and distance; bar 2 μm. (B) Quantification of the thickness of cell body and cell periphery of the non-silencing and β-PIX-silencing H1299 cells from the z-series images, as shown in (A). Data are mean ± SEM (*n* = 10 cells for each condition). ***P < 0.001, in comparison with non-silencing cells. (C) Epi-fluorescence images of non-silencing and β-PIX-silencing H1299 using staining with fluorescent phalloidin, to localize F-actin, and staining for DNase-I, to localize G-actin; bar 15 μm. (D) Ratio of the integrated intensity of F-actin normalized against G-actin. Data are mean ± SEM (*n* = 14 non-silencing cells; 10 β-PIX-silencing cells). **P < 0.01, in comparison with non-silencing cells. (E) Stiffness (Young's modulus; Pa) of non-silencing (*n* = 18 cells) and β-PIX-silencing (*n* = 25 cells) H1299 cells measured on the top of the nucleus and the cytosol, as described in Materials and methods. Data are mean ± SEM. **P < 0.01, as compared with non-silencing cells.

Next, we examined how β-PIX regulates actin cytoskeleton organization. Non-silencing and β-PIX-silencing H1299 cells were immunolabelled for F-actin and then imaged by optically slicing along the z-axis using 0.2 μm steps from top to bottom. The three-dimensional images were then reconstructed from the stack of two-dimensional images and these revealed that the peripheral regions of non-silencing cells contained a dense network of actin, while those of β-PIX-silencing cells contained a diffuse actin network (Fig.4A). We next examined the thickness of the cells in cell body and cell periphery, and observed that β-PIX-silencing H1299 cells had thinner cell periphery (Fig.4B), suggesting that the presence of β-PIX increased actin polymerization at cell periphery. To quantify the ability of β-PIX to promote actin polymerization, non-silencing and β-PIX-silencing H1299 cells were immunostained with phalloidin and DNase I, to visualize F-actin and G-actin, respectively, and imaged using epi-fluorescence microscopy (Fig.4C). Quantification of the ratio of the integrated fluorescence intensities of F-actin to G-actin in each cell indicated that increased β-PIX expression resulted in a significant increase in the ratio of F-actin to G-actin; hence, β-PIX appears to positively regulate actin polymerization.

As β-PIX has been linked to the actin polymerization (Fig.4C and D) 17,20,22 and the formation of dense actin networks at the peripheral region of H1299 cells (Fig.4A), we next investigated whether cell stiffness (Young's modulus) changed with the expression level of β-PIX. The Young's modulus (Pa) of non-silencing and β-PIX-silencing H1299 cells was measured using AFM. In the AFM measurement, the indentation surface was supposed to be a local measurement, so we applied the 5 μm diameter bead-conjugated cantilever for the stiffness measurement on the top of the cell nucleus and the cytosol. The stiffness measured on the top of nucleus represented the stiffness of nucleus, cell membrane and cytoskeleton cover on the top of nucleus, while the stiffness measured on the top of cytosol indicated the stiffness of the regions among nucleus and cell edge, including the cytosolic fluid and the cytoskeletons crossed by. We found that silencing of β-PIX significantly softened the regions measured on the top of the nucleus and the cytosol of a H1299 cell (Fig.4E), supporting that β-PIX regulated the actin cytoskeleton architecture because of the increase in actin polymerization (Fig.4C and D). Evidence indicates that high cell deformability is a reliable biomarker of metastatic potential in many cancer cells 33–36, and we further confirmed that, in the highly metastatic H1299 cells, β-PIX stiffened local regions of a cell because of spatial heterogeneity of cellular cytoskeleton organization. These findings support the hypothesis that β-PIX expression level in living H1299 cells is correlated with their viscoelastic properties.

### β-PIX controls the intracellular viscoelasticity gradient in migrating H1299 cells

**Figure 5:**
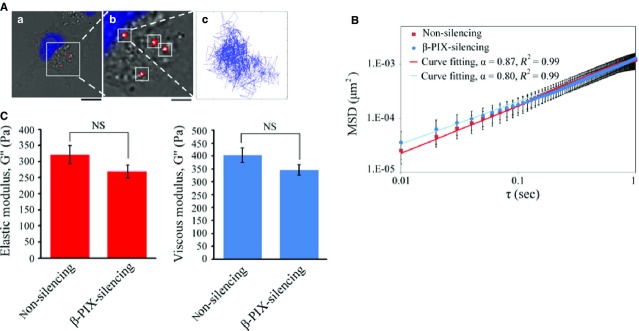
Multiple-particle tracking in non-silencing and β-PIX-silencing H1299 cells. (A) Typical trajectories of 100 nm diameter fluorescent carboxylated polystyrene beads (red) embedded in the cytoplasm of H1299 cells with the nucleus was labelled in blue (a and b). Beads were delivered into the cells; and after overnight incubation, the two-dimensional Brownian motion of the intracellular fluorescent beads was tracked and recorded at 10 msec. temporal resolution and 20 nm spatial resolution for 10 sec. (a) Bar 15 μm; (b) bar 5 μm. (B) The mean squared displacement (MSDs) as a function of the time lag (τ) of the imbedded fluorescent particles in non-silencing and β-PIX-silencing H1299 cells. The MSDs were fit to the power law <Δr^2^(τ)> = Aτ^α^. Data are mean ± SEM (non-silencing, *n* = 201 beads/35 cells; β-PIX-silencing, *n* = 513 beads/45 cells). (C) Intracellular elastic modulus (G′) and viscous modulus (G″) of non-silencing and β-PIX-silencing H1299 cells at 10 Hz (non-silencing, *n* = 201 beads/35 cells; β-PIX-silencing, *n* = 513 beads/45 cells). Data are mean ± SEM. NS, no significance.

To confirm this hypothesis, we used video particle tracking microrheology (VPTM) 27,28,37–40 to compare the elastic and viscous moduli of non-silencing and β-PIX-silencing H1299 cells. In this experiment, 100 nm diameter fluorescence beads were ballistically injected into H1299 cells using a biolistic particle delivery system 27,41; this dispersed the beads throughout the cytoplasm and the beads in each cell were tracked using high-resolution video-microscopy (Fig.5A). On the basis of the ensemble-averaged MSD, we note that in all cases, the extent of the particles' Brownian motion during each measurement period is in the order of 0.35 μm, and the square root of MSD is typically in the range of 10^−2^ to 10^−1^ μm. Hence, not only the particle size (100 nm) but also the extent of their motion, is much smaller than the cell thickness (Fig.4B). The effect of finite cell thickness on the results of our particle tracking microrheology is thus negligible. In addition, as the diameter of bead (100 nm) was significantly larger than the actin cytoskeletal mesh size (∽20–40 nm) 42, the Brownian motion of these beads reflects the viscoelastic properties of the actin cytoskeletal network in which they are embedded. In Figure5B, the MSD did not show significant differences between non-silencing and β-PIX-silencing H1299 cells. Next, we calculated the intracellular elastic modulus, G′, and viscous modulus, G″, of non-silencing and β-PIX-silencing H1299 cells at a frequency f of 10 Hz using the recently derived nematic pseudo-Stokes Einstein relation 27,40,43,44. Our results indicated that β-PIX did not significantly change the elastic modulus (G′) and the viscous modulus (G″; Fig.5C). However, we noticed that dense actin network induced by β-PIX was localized, specifically it was present close to the cell membrane (Fig.4A), which suggests that the β-PIX-mediated viscoelastic properties of living H1299 cells might be heterogeneous across the cell.

**Figure 6:**
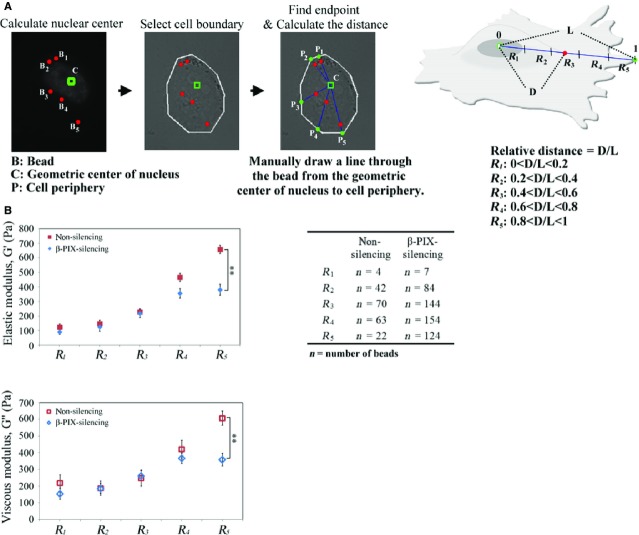
β-PIX spatially regulates cellular viscoelasticity. (A) Workflow for categorizing the location of fluorescent beads into five regions (R1, R2, R3, R4, and R5) based on their relative distance with respect to the geometric centre of nucleus and cell periphery. R1 is the region close to the geometric centre of nucleus; R5 is the region close to cell periphery. (B) Comparison of the elastic moduli and viscous moduli as a function of the relative distance outline in (A). Data are mean ± SEM (*n* for each conditions is indicated in the table). **P < 0.01, in comparison with non-silencing cells.

To test this hypothesis, we grouped the Brownian particles into five populations (R1, R2, R3, R4, R5) based on their intracellular location, as shown in Figure6A, and compared the elastic and viscous moduli of these populations in non-silencing and β-PIX-silencing H1299 cells. We found that, in the R5 area, the relative changes in elastic and viscous moduli were significantly lower in the β-PIX-silencing H1299 cells compared to the non-silencing cells. This indicates that the viscoelastic properties of the R5 area of a migrating H1299 cell are dependent of the level of β-PIX expression (Fig.6B). The differences in spatial viscoelasticity in terms of dependence on the level of β-PIX expression be explained in terms of β-PIX's function, namely that it is involved in the reconstitution of the actin cytoskeletal network at the leading edge of a migrating H1299 cell.

### Myosin II ATPase regulates viscoelastic actin networks in the cortex of a migrating H1299 cell

**Figure 7:**
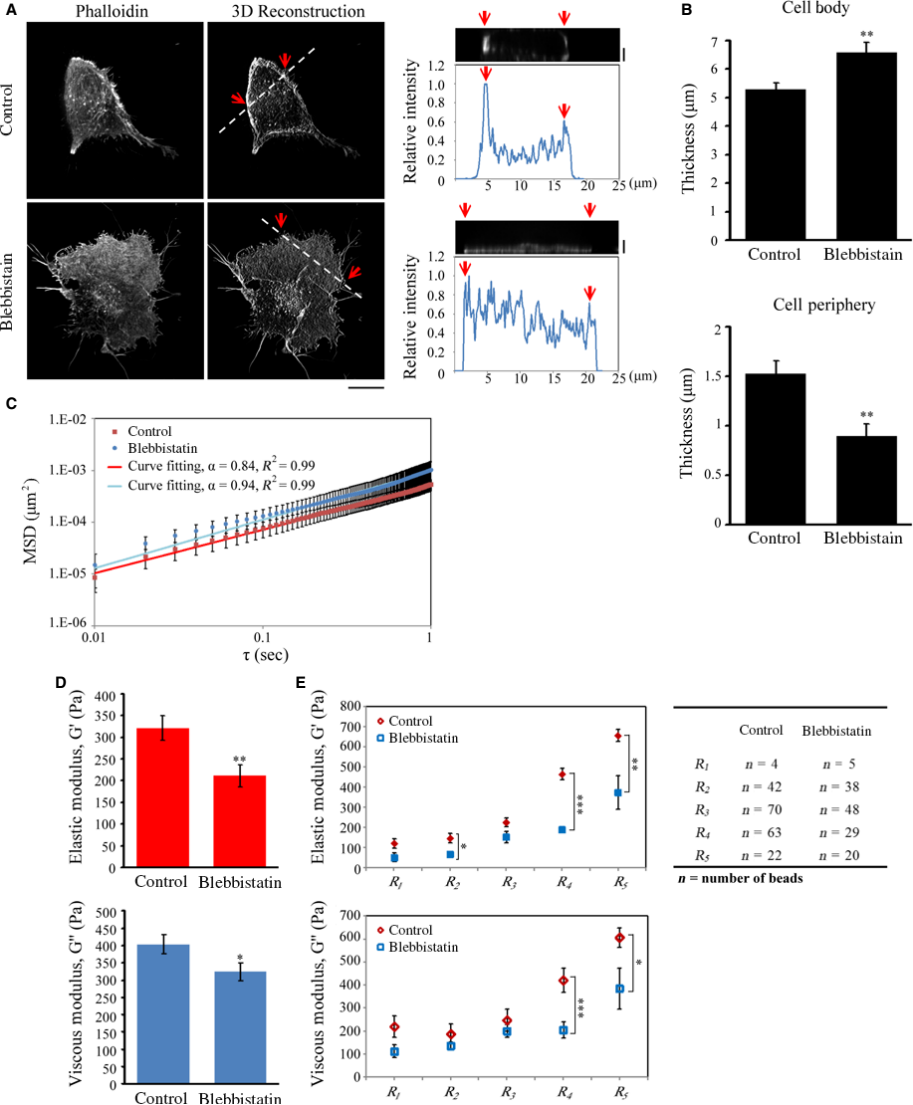
Inhibition of myosin II ATPase activity suppresses the organization of actin architecture and intracellular viscoelasticity at the cell periphery. (A, left) Confocal images of H1299 cells treated with control (DMSO) or blebbistatin (50 μM, 2 hrs) stained with fluorescent phalloidin to visualize F-actin. (Middle) 3D reconstruction taken from the z-series every 0.2 μm depth from top to bottom using confocal images of H1299 cells treated with control (DMSO) or blebbistatin (50 μM, 2 hrs) stained with fluorescent phalloidin; bar 15 μm. (Right) The side view image and relative fluorescent intensity as a function of position along the line highlighted in the 3D reconstruction image with the edges marked with red arrows; bar 2 μm. (B) Quantification of the thickness of cell body and cell periphery of H1299 cells treated with control (DMSO) or blebbistatin (50 μM, 2 hrs) from z-series images, as shown in (A). Data are mean ± SEM (*n* = 10 cells for each condition). **P < 0.01. (C) The mean squared displacement (MSD) as a function of the time lag (τ) of imbedded fluorescent particles in H1299 cells treated with control (DMSO) or blebbistatin (50 μM, 2 hrs). The MSDs were fit to the power law <Δr^2^(τ)> = Aτ^α^. Data are mean ± SEM (control, *n* = 201 beads/35 cells; blebbistatin, *n* = 140 beads/24 cells). (D) Elastic moduli and viscous moduli of H1299 cells treated with control (DMSO) or blebbistatin (50 μM, 2 hrs). Data are mean ± SEM (control, *n* = 201 beads/35 cells; blebbistatin, *n* = 140 beads/24 cells). **P < 0.01. (E) Comparison of the local elastic moduli and viscous moduli in five regions (R1, R2, R3, R4 and R5, as prescribed in Fig.6A) and deduced from the analysis of the Brownian motion of the beads in those regions. Data are mean ± SEM (*n* in each conditions is indicated in the table). *P < 0.05, **P < 0.01, ***P < 0.001, in comparison with non-silencing cells.

As myosin II ATPase inhibition in H1299 cells has been shown to have a similar effect to the silencing of β-PIX based on random migration analysis (Fig.2), this implies that, in H1299 cells, myosin II ATPase activity might be able to regulate actin cytoskeleton organization and intracellular viscoelastic actin networks. To test this hypothesis, we next examined the role of myosin II ATPase activity in regulating actin cytoskeleton organization. After 2 hrs of control (DMSO) or blebbistatin treatment, the cellular organization of F-actin networks were imaged by spinning disk confocal microscopy in the z-series using 0.2 μm steps from top to bottom. The z-series images were reconstructed into three-dimensional images and these showed a less-concentrated actin network in the blebbistatin-treated H1299 cells (Fig.7A). This supports the hypothesis that myosin II ATPase activity is required for the organization of actin networks in the cortex of cells.

Next, we examined whether myosin II ATPase activity regulates the intracellular viscoelastic properties of H1299 cells. The cell thickness (Fig.7B) and MSD (Fig.7C) were measured to examine the possible effect on microrheology measurement using VPTM. We found that both the particle size (100 nm) and their motion (the sqrt(MSD)) are much smaller than the cell thickness (Fig.7B); hence, so the particle tracking microrheology method was not affected by finite cell thickness. As we described earlier, the fluorescence beads were dispersed throughout the cytoplasm using a biolistic particle delivery system. The two-dimensional motion of individual beads in each cell was then tracked using high-resolution video-microscopy. Evaluation of the elastic and viscous moduli at 10 Hz indicated that the values were significantly reduced after blebbistatin treatment (Fig.7D). To further determine whether myosin II ATPase activity regulates intracellular viscoelastic properties spatially, the particles were analysed based on their intracellular location as described previously (Fig.6A). Figure7E showed that blebbistatin treatment significantly suppressed the elastic (G′) and viscous (G″) moduli in the R4 and R5 areas, indicating that myosin II ATPase activity is able to control viscoelastic properties in the R4 and R5 areas of migrating H1299 cells. When taken together, these findings suggest that, in a migrating H1299 cell that was isolated from a secondary lung cancer of the lymph nodes, myosin II ATPase activity contributes to the regulation of the reconstructed actin cytoskeleton networks and to the viscoelastic properties of the cortex.

## Discussion

**Figure 8:**
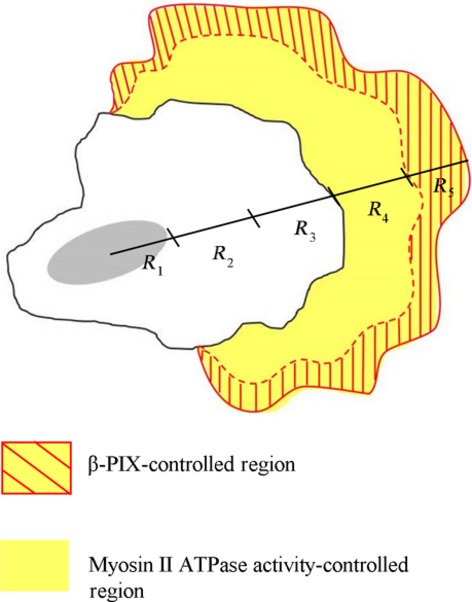
A model showing β-PIX-mediated signalling and myosin II ATPase-mediated signalling spatially controlling intracellular viscoelasticity and actin cytoskeleton organization, both of which contribute to the coordination of the cell migration process in non-small cell lung adenocarcinoma cells. In a highly metastatic lung cancer cell isolated from a secondary lung cancer of the lymph nodes, namely H1299 cells, the β-PIX-mediated signals are fully activated, as the cells lack bundled actin filaments (stress fibres). β-PIX mainly is localized at the immature FAs that drive lamellipodia extension, enhancing the integrity of the actin networks, up-regulating intracellular viscoelasticity at the cell periphery (R5 region), and control cell polarity, which then promotes directed cell migration. When compared to the cells with stress fibres, our findings show that there are a variety of different functions for myosin II ATPase, including promoting the organization of the actin cytoskeleton, changing intracellular viscoelasticity in the R4 and R5 regions, and modulating cell migration. For details, see Discussion.

Our study has uncovered a role for β-PIX and myosin II ATPase activity in regulating cortical actin structures that contribute to the intracellular viscoelasticity and mobility of non-small cell lung adenocarcinoma cells isolated from a secondary lung cancer of the lymph nodes, namely H1299 cells. Unlike the assumptions underlying the original model, in which the activity of β-PIX is assumed to be restrained by interacting with myosin II heavy chains in stress fibres, and is switched on when there is inhibition of myosin II ATPase, which disrupts stress fibres and releases β-PIX 22. In the present study, we have found that, in H1299 cells, β-PIX is not regulated by myosin II ATPase activity, and plays a direct regulatory role in promoting dense actin networks, intracellular viscoelasticity to stiffen cortical region (R5 region), cell polarity and directed cell migration, supporting the notion that β-PIX serves as a GEF protein with activity towards Rac1 and Cdc42 19,20,45. However, surprisingly, myosin II ATPase activity does also appear to play a similar role to that of β-PIX in that it helps to maintain the dense network of actin filaments present in cell cortex, although the region controlled by myosin II ATPase activity (the R4 and R5 regions) is wider than that regulated by β-PIX signalling (the R5 region only; Fig.8). Here, we have demonstrated for the first time that the mobility of a non-small cell lung adenocarcinoma cell is related to their structure of bundled actin filaments, which controls the signals of β-PIX.

Our results suggest a model for non-small cell lung adenocarcinoma cells isolated from a secondary lung cancer of the lymph nodes, H1299 cells, in which β-PIX mediates the progress of maturating nascent adhesions to focal complexes. This supports the notion that β-PIX main function is to maintain FAs in immature state 17,23. The maturation process from focal complexes to mature FAs has been found to be correlated with the myosin II-mediated contractile force, which is transduced along the bundled actin filaments (stress fibres) 46. Therefore, the lack of stress fibres in H1299 cells is mirrored by the loss of mature FAs (Fig.3B–D). Nevertheless, our results show that the number of nascent adhesions is increased in β-PIX-silencing H1299 cells (Fig.3D), which suggests a mechanism whereby the signalling by β-PIX is dictated by its localization, which then is able to mediate focal complexes formation and enhance cell migration.

Our study also reveals a previously unrecognized role for β-PIX in the spatial regulation of cell microrheology. This takes place in the physiologically relevant context of the mobility of non-small cell lung adenocarcinoma cells. We have shown that β-PIX is able to control the mechanical properties of H1299 cells by modulating the organization of the dense actin networks present in the cortex (Fig.4A). This observation identifies a function of β-PIX in promoting actin polymerization within the cell (Fig.4C and D). The organization of a reconstituted actin filament network has been linked to the motion of particles embedded in the actin cytoskeleton structures in living cells, which in turn leads to a reinforcement of the elasticity and viscosity of the cytoskeleton 28,47,48. Our measurements, *via* VPTM (Figs5 and 6), show that higher expression of β-PIX does indeed spatially mediate intracellular viscoelasticity of H1299 cells, which supports the hypothesis that β-PIX signalling contributes to local changes in cytoskeletal elasticity and viscosity and helps to bring about indispensable events associated with cell migration, which includes pushing forces, pulling forces and changes in cell shape.

In addition, our results, when taken together with previous studies, also support the hypothesis that the regulation of β-PIX's function is restrained by sequestering by the bundled actin filaments (stress fibres) 22. Evidence indicates that high cell deformability is a biomarker of metastatic potential in many types of cancer cells 33–36. In the highly metastatic non-small cell lung adenocarcinoma cell line H1299, we have shown that the lack of stress fibres results in localization of β-PIX to FAs, as well as an increase in actin polymerization, both of which promote lamellipodia extension and increased cell mobility. Therefore, the formation of stress fibres may result in their interaction with β-PIX and this in turn lead to a suppression of cell motility. However, the mechanism whereby there is a loss of stress fibres in the cells isolated from a secondary lung cancers of the lymph nodes remains unknown. Future studies are clearly needed to help clarify the above important questions.

## Supporting Information

**Video S1** Movies of H1299 cells expressing F-tractin-GFP and mApple-paxillin showing the dynamics of F-actin and FAs, respectively. Bar 15 μm.

**Video S2** Movies showing the migratory behaviour of non-silencing and b-PIX-silencing H1299 cells to b-PIX-silencing H1299.
